# Common themes in PrP signaling: the Src remains the same

**DOI:** 10.3389/fcell.2014.00063

**Published:** 2014-10-28

**Authors:** Katharina Ochs, Edward Málaga-Trillo

**Affiliations:** Department of Biology, University of KonstanzKonstanz, Germany

**Keywords:** prion protein, Src family kinases, ion channels, myelination, tyrosine phosphorylation, endocytosis, knockout mice, zebrafish

## Abstract

The ability of the cellular prion protein (PrP^C^) to trigger intracellular signals appears central to neurodegeneration pathways, yet the physiological significance of such signals is rather puzzling. For instance, PrP^C^ deregulation disrupts phenomena as diverse as synaptic transmission in mammals and cell adhesion in zebrafish. Although unrelated, the key proteins in these events -the NMDA receptor (NMDAR) and E-cadherin, respectively- are similarly modulated by the Src family kinase (SFK) Fyn. These observations highlight the importance of PrP^C^-mediated Fyn activation, a finding reported nearly two decades ago. Given their complex functions and regulation, SFKs may hold the key to intriguing aspects of PrP biology such as its seemingly promiscuous functions and the lack of strong phenotypes in knockout mice. Here we provide a mechanistic perspective on how SFKs might contribute to the uncertain molecular basis of neuronal PrP phenotypes affecting ion channel activity, axon myelination and olfactory function. In particular, we discuss SFK target proteins involved in these processes and the role of tyrosine phosphorylation in the regulation of their activity and cell surface expression.

Yes, there are two paths you can go by but in the long run There's still time to change the road you're on

(R. Plant and J. Page)

## Introduction

The abnormal accumulation of misfolded proteins is a defining molecular landmark of neurodegenerative conditions like prion, Alzheimer's and Parkinson's diseases. Interestingly, the identity of the pathogenic protein is different in each of these illnesses, suggesting that neurotoxicity may result from the excess of virtually any rogue protein in neuronal tissue. On the other hand, at least some of these proteins may play physiological roles in their non-aggregated state, which are key to neuronal survival and disease. For instance, the amyloid precursor protein and α-synuclein—the protein culprits of Alzheimer's and Parkinson's disease—are enriched in neuronal synapses and nerve terminals (Maroteaux et al., 1988; Schubert et al., 1991). Likewise, expression of PrP^C^ on neuronal cell surfaces is required for pathogenic prions and aβ oligomers to trigger cellular damage (Chesebro et al., 2005; Um et al., 2012). Beyond their propensity to misfold and aggregate, little is known about how exactly these proteins contribute to neuronal physiology and disease. For PrP, the quest for a gene/protein function has been particularly challenging because of the paucity of clear knockout phenotypes in mice (Steele et al., 2007). While this phenomenon may be explained by compensatory mechanisms (Málaga-Trillo and Sempou, 2009), the actual extent to which other genes can functionally replace PrP is only partly understood (Passet et al., 2013). More recent analyses of PrP knockout mice have revealed subtle defects in axon myelination and olfactory function as well as in the proliferation of neural precursors and self-renewal of hematopoietic stem cells (Steele et al., 2006; Zhang et al., 2006; Le Pichon et al., 2009; Bremer et al., 2010). Unfortunately, the precise molecular basis of these phenotypes remains unclear. Adding complexity to the matter, many of the dissimilar functions and interaction partners proposed for PrP^C^ cannot easily be accounted for by a single biological activity.

Among the various molecular roles ascribed to PrP^C^, its ability to elicit intracellular signals is in good correspondence with its cell surface localization and involvement in physiological processes as diverse and complex as cell adhesion, lymphocyte activation, neuroprotection, and synaptic function (Aguzzi et al., 2008). That PrP signaling is pathophysiologically relevant was elegantly shown by Chesebro and colleagues, who demonstrated that without anchoring to the plasma membrane, PrP may misfold and aggregate but not induce neuronal damage (Chesebro et al., 2005). Presently, a number of transmembrane and intracellular molecules are known to help PrP^C^ transduce signals into the cell's interior (Linden et al., 2008). Among the latter, SFKs have gathered renewed interest because of their connection to prion- and aβ-induced neurotoxicity as well as for their contribution to PrP-mediated cell communication *in vivo*. The purpose of this article is to revisit putative functions of PrP in the context of SFK signaling.

## SFKs, a multifunctional protein family

The Src family of non-receptor protein tyrosine kinases comprises nine members in vertebrates, five of which are active in mammalian CNS neurons, namely the ubiquitously expressed Src, Fyn, and Yes, and the tissue-restricted Lck and Lyn (Thomas and Brugge, 1997; Salter and Kalia, 2004). Their association to the cytoplasmic face of the plasma membrane via myristoylation and palmytoylation enables them to receive extracellular stimuli and transduce them into the cell interior (Silverman et al., 1993). SFK enzymatic activity is triggered by autophosphorylation at tyrosine residue 416 and inhibited by phosphorylation of tyrosine residue 527 (Roskoski, 2005). Owing to their interaction with a diverse array of transmembrane receptors and downstream targets, SFKs can regulate a broad range of cellular processes, including cell differentiation, proliferation, adhesion, migration, apoptosis, and immunity (Thomas and Brugge, 1997; Roskoski, 2004). Because of their strong structural conservation, SFK members are functionally redundant and can often compensate for each other, encumbering analysis of their individual roles via gene knockout approaches (Lowell and Soriano, 1996). Given their expression and multiple functions in neurons, it is not surprising that SFK deregulation should contribute to neurodegeneration. In fact, prion infection triggers increased levels of Src and phosphotyrosine proteins in cultured cells and mice (Nixon, 2005; Gyllberg et al., 2006). Moreover, Fyn is selectively upregulated in Alzheimer's brains (Shirazi and Wood, 1993), where it mediates hyperphosphorylation of the aggregation-prone, microtubule-associated protein Tau (Lee et al., 2004). Fittingly, Fyn overexpression triggers synaptic damage in mouse models of Alzheimer's disease (Chin et al., 2005) whereas Fyn knockdown leads to decreased Tau phosphorylation, increased aβ levels and impaired spatial learning (Minami et al., 2012). Together, these findings imply a direct involvement of SFKs in neurotoxic pathways that are common to multiple neurodegenerative conditions.

## SFKs as functional hubs of PrP

Being tethered to the outer leaflet of the plasma membrane via a short glycosylphosphatidylinositol- (GPI) anchor, PrP is unlikely to physically associate with cytosolic SFKs. Nevertheless, co-immunoprecipitation data indicate that, in epithelial cells, PrP and SFKs interact at least indirectly within a larger protein complex (Morel et al., 2004, 2008). In addition, plenty of evidence supports the notion that PrP and SFKs are functional partners in neurons. Using antibody-mediated cross-linking, Mouillet-Richard and colleagues were the first to show that PrP clustering at neuronal cell surfaces triggers caveolin-dependent activation of Fyn (Mouillet-Richard et al., 2000). Related experiments in neuron-like PC12 cells corroborated the contribution of caveolin to PrP^C^-mediated Fyn activation, along with the downstream activation of the Ras-Raf/ERK pathway and the negative regulation of the entire cascade by phosphocaveolin (Pantera et al., 2009). In cultured mouse hippocampal neurons, PrP^C^ was found to indirectly activate Fyn via *cis* or *trans* associations with the neural cell adhesion molecule (NCAM) within lipid rafts, thereby stimulating neurite outgrowth (Santuccione et al., 2005). Importantly, *in vivo* data from a wide range of animal models underscore the relevance of the PrP/SFK pathway in physiology and disease. For instance, expression of a pathogenic PrP mutant in *Caenorhabditis elegans* induced neuronal dysfunction in a Fyn-dependent manner (Bizat et al., 2010), a remarkable result since nematodes do not possess endogenous PrPs. In early zebrafish embryos, exogenously added mouse PrP mimicks the positive regulation of E-cadherin-mediated cell adhesion by endogenous PrPs, an effect that requires Fyn and Yes activity (Málaga-Trillo et al., 2009; Sempou et al., submitted). In undoubtedly one of the most sensational developments in the recent field of neurodegeneration, neuronal PrP^C^ was reported to act as a receptor for aβ oligomers. Notably, the resulting synaptic impairment was mediated by Fyn (Um et al., 2012).

**Figure 1:**
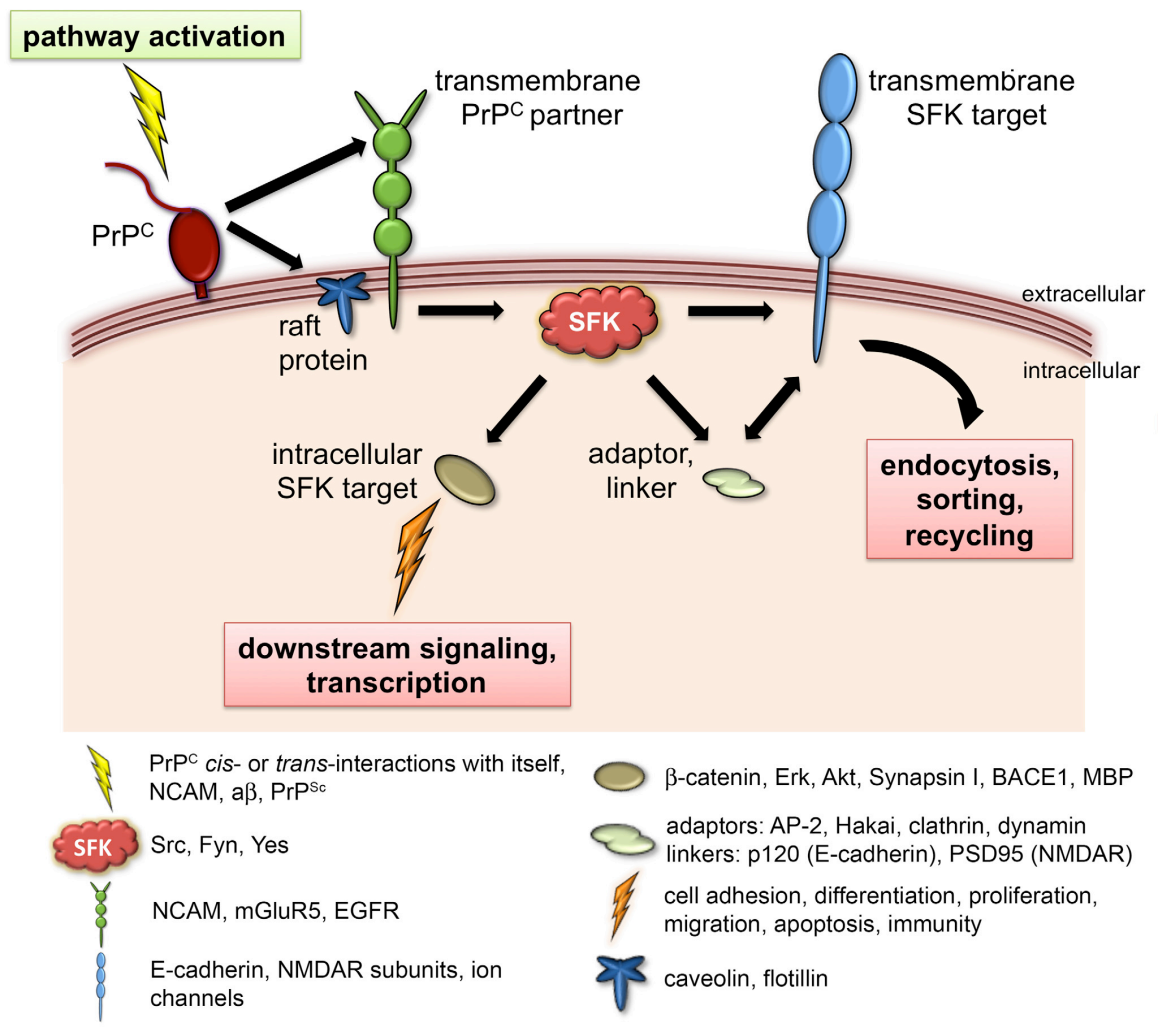
**PrP^C^ signaling via SFKs**. Known physiological roles of PrP^C^ such as neurotransmission, embryonic cell adhesion, olfactory function, and myelination converge at the use of SFKs as intracellular signaling partners. In this model, engagement of PrP^C^ in various *cis*- or *trans*-interactions (depicted as “pathway activation”) elicits the catalytic activity of SFKs, leading to the modulation of diverse downstream targets that include ion channels, adhesion complexes, and cytosolic signaling molecules. Phosphorylation of transmembrane SFK targets controls their cell surface expression and/or signaling properties, whereas phosphorylation of intracellular SFK targets regulates the activation of additional downstream pathways, gene transcription, and protein translation. The caption below provides concrete examples of documented components of this cascade.

At first glance, some of these findings have no apparent connection with each other, aside from the common involvement of SFKs. However, closer scrutiny reveals mechanistic similarities pertinent to our understanding of PrP function. This is particularly manifest in two of the aforementioned models, where the downstream targets of PrP turned out to be transmembrane proteins modulated by tyrosine phosphorylation. In the study by the Strittmatter lab, binding of aβ oligomers to PrP^C^ triggered Fyn activation and hyperphosphorylation of the NR2B subunit of the NMDAR at tyrosine residue 1472 (Um et al., 2012). As a result, NMDARs became overstabilized at the plasma membrane and glutamate excitotoxicity ensued. Under non-pathological conditions, phosphorylation of NR2B at this C-terminal regulatory site promotes its normal cell surface expression by preventing the binding of AP-2 adaptor complexes, which would otherwise initiate its internalization via clathrin- and dynamin-dependent endocytosis (Salter and Kalia, 2004). Similarly, postsynaptic density protein 95 (PSD-95) prevents NR2B endocytosis by binding a site adjacent to tyrosine 1472 and thereby blocking access to AP-2 (Lavezzari et al., 2003). Overall, the phosphorylation state of NMDAR subunits is controlled by the dynamic interplay between SFKs and protein tyrosine phosphatases (PTPs), as well as by the input from various upstream signaling molecules (Salter and Kalia, 2004). In addition, NMDAR function is further regulated via differential transcription, translation and trafficking of specific subunits as well as through protein-protein interactions (Sanz-Clemente et al., 2013). Notably, the regulation of protein endocytosis by molecules that competitively bind target motifs at cytoplasmic tails is also pivotal to the modulation of E-cadherin cell surface expression (Ishiyama et al., 2010). Thus, association of p120 catenin to the C-terminal juxtamembrane domain (JMD) of E-cadherin sterically blocks binding of AP-2 and the E3 ubiquitin ligase Hakai, thereby preventing its clathrin-mediated or ubiquitination-dependent endocytosis, respectively (Thoreson et al., 2000; Fujita et al., 2002; Miyashita and Ozawa, 2007; Ishiyama et al., 2010). Importantly, SFK-mediated phosphorylation of the JMD and/or p120 catenin triggers E-cadherin internalization (Reynolds, 2010). Additionally, SFKs phosphorylate and modulate components of the endocytic machinery like clathrin, dynamin and AP-2 (Ahn et al., 1999, 2002; Wilde et al., 1999; Zimmerman et al., 2009). In our zebrafish model, PrP^C^ promotes embryonic cell adhesion by stabilizing E-cadherin at the cell-surface (Málaga-Trillo et al., 2009). Crucially, this activity entails the SFK-dependent inhibition of E-cadherin endocytosis (Sempou et al., submitted). Therefore, the roles of PrP^C^ as a modulator of cell-cell adhesion in zebrafish and synaptic transmission in mammals converge at a common mechanism, namely the ability of SFKs to control the internalization of trans-membrane proteins by phosphorylating their endocytic signals and/or the corresponding binding molecules. Given the functional diversity of SFK target proteins, we reasoned that altered SFK activity may account for at least some of the distinct neuronal phenotypes observed in PrP knockout and transgenic mice (Figure 1, Table 1). To address this possibility, we examined three compelling, yet poorly understood paradigms of PrP function.

**Table 1:** **SFKs and downstream events involved in PrP activities**.

**PrP activities reported**	**SFKs involved**	**SFK target events affected**	**References**
aβ oligomer neurotoxicity	Fyn	Endocytosis of NMDA receptor subunit NR2B	Salter and Kalia, 2004
Regulation of embryonic cell adhesion (zebrafish)	Fyn, Yes	Endocytosis of E-cadherin	Ishiyama et al., 2010
PrPΔCR neurotoxicity	Fyn	Gating and/or endocytosis of NMDARs	Salter and Kalia, 2004
PrP PG14 neurotoxicity	Src	Gating and/or endocytosis of VGCCs	Davis et al., 2001
Regulation of olfactory physiology	Src	Binding of Synapsin Ib to synaptic vesicles and actin	Messa et al., 2010
	Src	Gating and/or trafficking of K_v_1.3 channels	Fadool and Levitan, 1998; Fadool et al., 2000
	Fyn	Gating and/or endocytosis of NMDARs	Chen et al., 2000; Halabisky et al., 2000
Molecular control of myelination	Src	Activation of Akt/PKB	Chen et al., 2001; Jiang and Qiu, 2003
	Src	Endocytosis and activation of BACE1	Zou et al., 2007
	Src	mTORC1-induced transcription of the MBP gene	Vojtechova et al., 2008; Ondrusova et al., 2013
	Fyn	Localized translation of MBP mRNA at axon–glia contact sites via hnRNP A2	White et al., 2008
	unknown	Endocytosis of MAG and MOG	Bo et al., 1995; Kroepfl and Gardinier, 2001a,b
	Src, Fyn	Gating and/or endocytosis of K_v_1.5 and 2.1 channels	Sobko et al., 1998; Peretz et al., 1999; Tiran et al., 2006

*Summary of some previously described activities of PrP (see main text) and the relevant molecular targets/events regulated by SFKs. The actual involvement of these pathways in the corresponding PrP phenotypes is hypothetical, except for those under “aβ oligomer neurotoxicity” and “embryonic cell adhesion,” which have been experimentally confirmed*.

### PrP and ion channels

The NMDAR-mediated glutamate excitotoxicity induced by aβ oligomers and PrP^C^ clearly shows that the latter can influence the activity of ion channels (Um et al., 2012). Intriguingly, while this effect relies on the PrP-dependent overactivation of NR2B subunits, a different study found that PrP^C^ suppresses NMDAR activity and glutamate excitotoxicity via the inhibition of NR2D subunits (Khosravani et al., 2008). How PrP may influence this pathway under physiological conditions remains to be established. Nonetheless, a general role of PrP as a modulator of ion channel activity and neurotransmission is consistent with its synaptic localization (Moya et al., 2000; Um et al., 2012) and the common occurrence of electrophysiological alterations in PrP knockout mice (Aguzzi et al., 2008; Biasini et al., 2012). Moreover, transgenic mice expressing neurotoxic PrP mutants show distinct neurological phenotypes related to ion channel dysfunction. One of these mutants, PrPΔCR, has 21 aa deleted from its central region and triggers neonatal lethality characterized by the spontaneous degeneration of cerebellar granule neurons (CGNs) (Li et al., 2007). Subsequent work in cultured cells and cerebellar slices revealed that PrPΔCR induces spontaneous currents that increase neuronal susceptibility to glutamate-dependent, Ca^2+^-mediated excitotoxicity (Solomon et al., 2010; Biasini et al., 2013). Based on these data, the authors suggested that PrPΔCR might either activate an endogenous ion channel or form itself an ion channel. A second PrP mutant, PG14, causes age-related degeneration of CGNs owing to defective glutamate exocytosis and impaired calcium dynamics (Senatore et al., 2012). This phenotype was explained by the ability of PG14 to bind the voltage-gated calcium channel (VGCC) α_2_δ-1 subunit and retain it at the ER, thus disrupting its delivery to the plasma membrane. Notably, while the molecular mechanisms of these two phenotypes seem unrelated, they are both consistent with the ability of SFKs to regulate the activity of ligand- and voltage-gated ion channels (Salter and Kalia, 2004). For instance, the spontaneous ionic currents induced by PrPΔCR may be explained by alterations in SFK-mediated phosphorylation and trafficking of NMDAR subunits. Detailed biochemical analyses will be needed to distinguish between this scenario and one proposed by the authors, namely, that the mutant PrP molecule activates NMDARs via direct interaction (Biasini et al., 2013). For the PG14 phenotype, the data show that intracellular retention of VGCC α_2_δ-1 during biosynthesis is the most likely explanation for its reduced levels at the plasma membrane (Senatore et al., 2012). However, here it also might be worthwhile considering that VGCCs are modulated via tyrosine phosphorylation by, among others, Src (Davis et al., 2001). Whether SFK regulation of VGCCs targets their trafficking or only their gating properties is presently unknown.

### PrP and olfaction

Unique among mouse PrP knockout phenotypes was the finding of impaired olfactory behavior (Le Pichon et al., 2009). In electrophysiological terms, the phenotype was attributed to olfactory bulb-specific alterations in the patterns of oscillatory activity and in the plasticity of the dendrodendritic synapse. No molecular basis was reported for these defects but the authors suggested that due to its localization at olfactory synaptic membranes and its interaction with Synapsin Ib (Spielhaupter and Schatzl, 2001), PrP may be a regulatory component of the synaptic machinery. Interestingly, several lines of evidence argue for a contribution of SFKs and their targets to this phenotype. First, phosphorylation of Synapsin I by Src increases its binding to synaptic vesicles (SVs) and the actin cytoskeleton, thereby limiting the number of recycled SVs available for exocytosis and neurotransmitter release (Messa et al., 2010). Second, Fyn-deficient mice exhibit defects related to the olfactory dysfunction described by Le Pichon et al., such as impaired suckling behavior, altered synaptic transmission via GABA and NMDA receptors in the olfactory bulb, and abnormal axon guidance and fasciculation in the olfactory nerve (Yagi et al., 1993; Kitazawa et al., 1998; Morse et al., 1998). Third, K_v_1.3 voltage-gated potassium channels are expressed along the dendrodendritic synapse and are negatively regulated by Src phosphorylation (Fadool and Levitan, 1998; Fadool et al., 2000). Remarkably, olfactory neurons from K_v_1.3 null mice have modified action potentials and deregulated potassium currents, resulting in structurally altered olfactory bulbs and a heightened sense of smell (Fadool et al., 2004). Fourth, NMDARs play a crucial role at the dendrodendritic synapse by generating the Ca^2+^ influx that triggers inhibitory GABA release (Chen et al., 2000; Halabisky et al., 2000). This essential component of olfactory learning is therefore also subject to modulation by PrP and SFKs. Hence, the combined, SFK-mediated deregulation of Synapsin I, K_v_1.3 channels, and NMDARs provides testable explanations for the olfactory defects of PrP knockout mice.

### PrP and myelination

Re-examination of a mild, late-onset phenotype in PrP knockout mice led to the discovery of defects in the maintenance of peripheral myelin (Bremer et al., 2010). This chronic demyelinating polyneuropathy (CDP) was triggered by the loss of PrP in neurons but not in Schwann cells. The mechanistic basis of this phenotype remains unidentified, as the authors found no alterations in the phosphorylation of Akt, and Erk, two signaling molecules down the myelination pathway initiated with the cleavage of Neuregulin-1 (NRG1) by the BACE1 endopeptidase (Hu et al., 2006; Liu et al., 2007). Similarly, no differences were detected in the expression levels of NRG1, although, neither BACE1 activity nor BACE1-mediated cleavage of NRG1 was directly assessed in PrP knockout mice. Interestingly, Src is known to modulate several events along the BACE1/NRG1 pathway. Some of them, like Akt phosphorylation by Src (Chen et al., 2001; Jiang and Qiu, 2003), are probably not involved in the PrP phenotype, as implied by Bremer and colleagues. Others like BACE1 endocytosis and activation (Zou et al., 2007) or the mTORC1-induced transcription of the myelin basic protein (MBP) gene (Vojtechova et al., 2008; Ondrusova et al., 2013) would require further verification. Of note, BACE-null mice exhibit myelination defects reminiscent of those in PrP knockout mice, as well as increased levels of full-length NRG1 and—unlike PrP knockouts—reduced Akt phosphorylation (Hu et al., 2006, 2008).

As with the olfactory phenotype of Le Pichon et al., copious evidence suggests a potential involvement of SFKs in the PrP-CDP phenotype. Comprehensive reviews on the role of Fyn during myelination are available elsewhere (Kramer-Albers and White, 2011; White and Kramer-Albers, 2014) but for example, myelination requires Fyn activation by the myelin-associated glycoprotein (MAG), and activated Fyn promotes MBP gene transcription (Umemori et al., 1994, 1999). In addition, Fyn stimulates the localized translation of MBP mRNA at sites of axon–glia contact via phosphorylation of the heterogeneous nuclear ribonucleoprotein (hnRNP) A2 (White et al., 2008). Furthermore, Fyn-deficient mice show myelination defects similar to those of BACE- and PrP-null mice, albeit with some differences in the localization (PNS vs. CNS), onset (late vs. early) and severity of the phenotype (Umemori et al., 1994, 1999; Sperber et al., 2001). Interestingly, the presence of tyrosine-based endocytic motifs in MAG and the myelin/oligodendrocyte glycoprotein (MOG) strongly suggests that their cell surface expression is modulated by SFKs (Bo et al., 1995; Kroepfl and Gardinier, 2001a,b). In fact, the endocytic trafficking and sorting of myelin proteins is key to the establishment of membrane domains in oligodendroglial cells (Winterstein et al., 2008). Particularly relevant to the PrP-CDP phenotype, adult and aged MAG-null mice undergo degeneration of myelinated fibers in the PNS (Schachner and Bartsch, 2000).

Finally, voltage- and ligand-gated ion channels constitute further regulatory targets of SFKs during myelination. For instance, the phosphorylation state of K_v_1.5 and K_v_2.1 potassium channels in Schwann cells is controlled by the interplay between Src, Fyn, PTPα, and PTPε (Sobko et al., 1998; Peretz et al., 1999; Tiran et al., 2006). This complex mechanism modulates the gating properties of the channels, influencing Schwann cell development and the onset of myelination. Likewise, NMDARs promote the maturation of oligodendrocyte precursor cells (OPCs) by enhancing the expression of myelin proteins (Li et al., 2013). Nevertheless, experiments with NMDAR-deficient mice suggest that, *in vivo*, this regulation might be relevant for re-myelination but not for OPC development (De Biase et al., 2011; Li et al., 2013). Thus, although the molecular control of myelination is a rather intricate phenomenon, the PrP-CDP phenotype seems consistent with the alteration of multiple pathways downstream of SFKs.

Altogether, the common regulation of ion channels, olfaction and myelination by PrP and SFKs suggests that this phenomenon may extend to some of the other subtle PrP phenotypes mentioned above. For instance, it would be interesting to see if the role of PrP^C^ in the proliferation and differentiation of various stem cell lineages (reviewed in Martin-Lannerée et al., 2014) requires the differential expression of SFKs and their targets at specific stages of stem cell maturation. Along these lines, the contribution of tissue-restricted SFKs to PrP signaling deserves further investigation, as most of the available data on the PrP/SFK pathway so far involve only the ubiquitously expressed Src, Fyn, and Yes.

## Conclusions

Several key observations emerge from the present analysis. First, the ability of PrP^C^ to elicit intracellular signals at the cell surface is central to its multiple roles in health and disease. Second, the specificity of these signals is largely determined by extrinsic factors such as the plasma membrane microenvironment and the availability of cell- and tissue-specific protein partners. Third, the ubiquitous expression and functional redundancy of SFKs, along with the diversity of their downstream targets offer plausible explanations for some of the striking facts of PrP biology, such as its functional promiscuity, the viability of PrP knockout mice, and the diversity of PrP transgenic/mutant phenotypes. Based on these considerations, we propose that PrP^C^ acts as a gatekeeper between neuronal survival and toxicity by controlling the activity and/or endocytic trafficking of ion channels, synaptic proteins and cell adhesion molecules via SFKs. Testing these complex scenarios should prove a formidable but rewarding endeavor.

### Conflict of interest statement

The authors declare that the research was conducted in the absence of any commercial or financial relationships that could be construed as a potential conflict of interest.
